# Molecular Machines in the Synapse: Overlapping Protein Sets Control Distinct Steps in Neurosecretion

**DOI:** 10.1371/journal.pcbi.1002450

**Published:** 2012-04-05

**Authors:** L. Niels Cornelisse, Evgeni Tsivtsivadze, Marieke Meijer, Tjeerd M. H. Dijkstra, Tom Heskes, Matthijs Verhage

**Affiliations:** 1 Functional Genomics, Center for Neurogenomics and Cognitive Research (CNCR), Department of Clinical Genetics, VUmc, Neuroscience Campus Amsterdam (NCA), VU University (VUA) and VU University Medical Center (VUmc), Amsterdam, The Netherlands; 2 Machine Learning Group, Intelligent Systems, Institute for Computing and Information Sciences, Radboud University, Nijmegen, The Netherlands; 3 Microbiology and Systems Biology Group, TNO, Zeist, The Netherlands; 4 Signal Processing Systems, Department of Electrical Engineering, Technical University Eindhoven, Eindhoven, The Netherlands; North Carolina State University, United States of America

## Abstract

Activity regulated neurotransmission shapes the computational properties of a neuron and involves the concerted action of many proteins. Classical, intuitive working models often assign specific proteins to specific steps in such complex cellular processes, whereas modern systems theories emphasize more integrated functions of proteins. To test how often synaptic proteins participate in multiple steps in neurotransmission we present a novel probabilistic method to analyze complex functional data from genetic perturbation studies on neuronal secretion. Our method uses a mixture of probabilistic principal component analyzers to cluster genetic perturbations on two distinct steps in synaptic secretion, vesicle priming and fusion, and accounts for the poor standardization between different studies. Clustering data from 121 perturbations revealed that different perturbations of a given protein are often assigned to different steps in the release process. Furthermore, vesicle priming and fusion are inversely correlated for most of those perturbations where a specific protein domain was mutated to create a gain-of-function variant. Finally, two different modes of vesicle release, spontaneous and action potential evoked release, were affected similarly by most perturbations. This data suggests that the presynaptic protein network has evolved as a highly integrated supramolecular machine, which is responsible for both spontaneous and activity induced release, with a group of core proteins using different domains to act on multiple steps in the release process.

## Author Summary

Synapses, which are small structures where information is transmitted between neurons, are the functional units of computation in the brain implicated in information processing, learning and memory. In the last few years several genes that are expressed in synapses have been linked to brain disorders such as autism, depression and schizophrenia. However, good understanding of the molecular basis of these diseases requires more insight in the functional organization of the protein network. In the classic view proteins are involved in a specific step of a cellular process, whereas modern system theories suggest a more integrated and complex network where proteins can be involved in several steps. We have developed a method that allows comparison of high-end but low-throughput functional data from different genetic perturbation studies despite the poor standardization between studies. Our analysis shows that synaptic proteins are not restricted to a single function but that they are part of a highly integrated supramolecular machine, with overlapping protein sets being involved in distinct steps in neurotransmitter release. Further characterization of these complex protein network relations will be important for new drug design strategies.

## Introduction

Synapses are complex biological structures, which evolved into highly specialized computational units that play an important role in learning, memory formation and information processing in the brain [1], [2], [3]. A key process in the synapse is the remarkably fast and precisely timed secretion of neurotransmitters from small synaptic vesicles in the nerve terminal upon arrival of an action potential (AP) [4]. In general, such complex cellular processes exist by virtue of the concerted action of many proteins, which are often found to sequester into multi-protein complexes, also referred to as molecular machines. It is common practice to assign specific proteins or sub-complexes to specific steps in a cascade of events. However, this intuitive idea has not been tested sufficiently. Gene knockout technology in mice and gene overexpression by viral constructs have been used in many studies for detailed functional analysis of individual mammalian proteins. However, systematic comparative analysis based on these studies is hampered by the fact that functional data from different studies are poorly standardized and therefore do not allow direct comparison of the observed perturbation effects as in less complex model organisms [5], [6], [7], [8], [9].

Over the past 15 years, the proteins that make up the synaptic release machinery have been largely identified [4], [10], [11]. Many of these were functionally studied in genetic perturbation studies using high-end but low-throughput assays. Here we present a novel probabilistic method to compare functional data from different studies and cluster perturbations of presynaptic genes according to their effect on different synaptic release parameters. The method addresses some fundamental problems in meta-analysis of poorly standardized functional data, such as large variation between different studies, incomplete reporting of strongly co-varying variables, and different effect sizes of perturbations, and was designed to be generally applicable to similar data sets in other experimental settings. We show that synaptic release is governed by a highly integrated molecular machine with a set of core proteins controlling both the steps of priming and fusion, implying that functionally distinct steps are not always regulated by distinct sets of proteins.

## Results/Discussion

### MPPCA allows clustering of functional data from different perturbation studies

**Figure 1:**
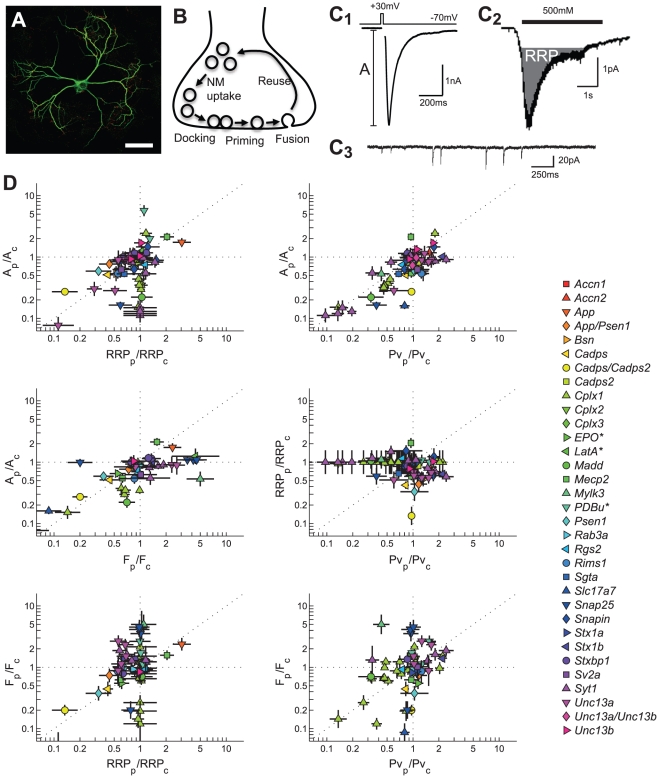
Functional data from genetic perturbation studies in autapses. A) Confocal image of an autaptic neuron, scale bar is 50 µm. B) Schematic representation of a synapse. In each synapse only a limited number of vesicles, referred to as the readily releasable pool (RRP), is in a primed state that allows direct fusion during calcium triggering. The probability that a primed vesicle fuses during a calcium trigger is given by the vesicular release probability (P_v_). C) Example traces of synaptic release variables. C1) Evoked EPSC (lower trace), upper trace shows somatic stimulation to trigger an action potential. C2) Postsynaptic response to a 500 mOsmol hypertonic stimulation for RRP estimation. C3) Spontaneous release events. D) Scatter plots of normalized and log-2 transformed synaptic variables in 2D projections of the 4D variable space for 121 perturbations (13 perturbations with >10-fold effect are not shown). *Non-genetic perturbations using a biochemical compound.

Vesicle priming and fusion, two important steps in the life cycle of synaptic vesicles, are the main determinants of synaptic strength. According to the quantal hypothesis of neurotransmitter release synaptic strength is given by
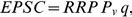
(1)with EPSC the excitatory post-synaptic current, RRP the size of the readily releasable pool, which contains all the vesicles that are primed to be released, P_v_ the vesicular release probability, which is the probability that a vesicle will fuse upon arrival of an action potential at the terminal, and q the postsynaptic quantal size, which is defined as the current response to the release of a single vesicle [12]. It is shown in several studies that manipulation of different presynaptic genes affect RRP size and P_v_. independently [13], [14], [15], [16]. To systematically compare the effect of presynaptic genes on vesicle priming and fusion we collected functional data from genetic perturbation studies in hippocampal island cultures (autapses) to reduce variation between datasets [17], [18] (Figure 1A). All data was obtained from genes with a known presynaptic gene product, transcription factors not included. We selected synaptic variables that were frequently enough reported among different studies to allow systematic comparison and retrieved data for 378 experiments (perturbations and control experiments) from 56 published studies (see database in Table S1). Included variables were the number of primed vesicles (RRP), the fusion probability (P_v_), the amount of evoked release (A), and the amount of spontaneous release (F) (Figure 1B–C). Since the variables RRP and P_v_ are expected to co-vary with A according to Eq. 1 most papers only report a subset of these. Wild-type data from different studies showed a large variation, with only A and P_v_, but not A and RRP, showing the linear correlation predicted by the release model (Suppl. Info. Figure S1). The variance could not be attributed to specific experimental conditions in the different studies, like the external calcium concentration or number of days in vitro (DIV) (Figure S2). Altogether, this shows that large variation prevents direct comparison between functional data of different studies. To overcome this problem and in addition allow comparison between different data formats (e.g. normalized vs. absolute values, different units) we normalized each perturbation to a control group from the same experiment and log2-transformed the data, yielding 121 normalized perturbations of 27 genes from 39 studies with two or more variables reported (Figure 1D).

**Figure 2:**
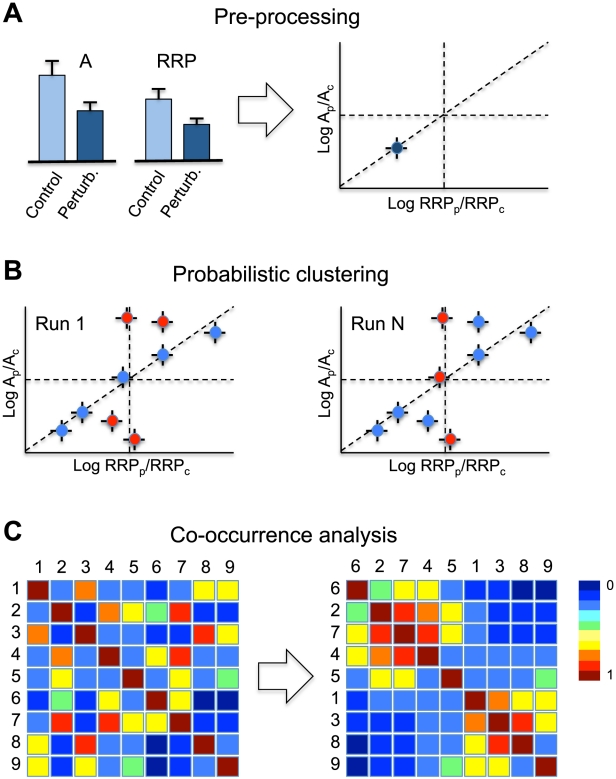
Schematic representation of data processing steps. A) Perturbations are extracted from publications then log-transformed and standardized relative to a control condition. B) Two exemplary runs of the probabilistic clustering algorithm for K = 2 clusters are shown in the left and right panels. Perturbations are colored red or blue depending on the cluster to which they are assigned with the highest probability. Note that some of the perturbations change color (cluster) for different runs of the probabilistic clustering algorithm. C) Co-occurrence analysis. By observing the co-occurrence of pairs of perturbations in the same cluster, we built a co-occurrence matrix with color indicating how often each pair co-occurs in the same cluster. In the right panel we have re-ordered the rows and columns to highlight the two dominant clusters in the data.

**Figure 3:**
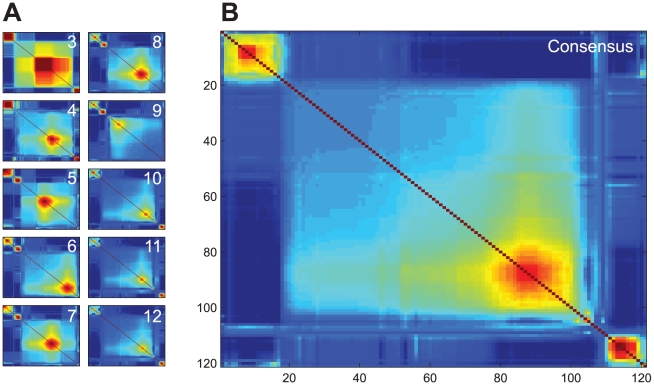
Co-occurrence matrices. A) Average over ten restarts of the co-occurrence matrices for clustering with a fixed number of clusters. Number of clusters K is indicated in each plot. Matrices were ordered for visualization using optimal leaf ordering. B) Consensus co-occurrence matrix obtained by averaging the individual co-occurrence matrices presented in A. Order of perturbations in the consensus matrix is given in Table S2. Red color denotes high co-occurrence, and blue low co-occurrence.

We designed a novel method to cluster perturbations with similar qualitative effects on vesicle release variables A, RRP, and P_v_, irrespective of different effect sizes for different perturbation types (e.g. heterozygous vs homozygous knock-out, mild vs strong overexpression), different experimental conditions, or missing variables. To this end we used a mixture of probabilistic principal component analyzers (MPPCA), which clusters the orientations of the normalized Log-2 transformed data in 3D variable space and assigns probabilities to the perturbations to belong to each of the detected clusters (for schematic overview see Figure 2, for a detailed description see Suppl. Info). Running the cluster algorithm revealed three clusters based on the structure of the average co-occurrence matrix, with 87, 21 and 13 perturbations assigned with the highest probability to cluster 1, 2 and 3, respectively (Figure 3, Suppl. Info. Figure S3, Table S2).

### Perturbation clusters reflect distinct synaptic function

**Figure 4:**
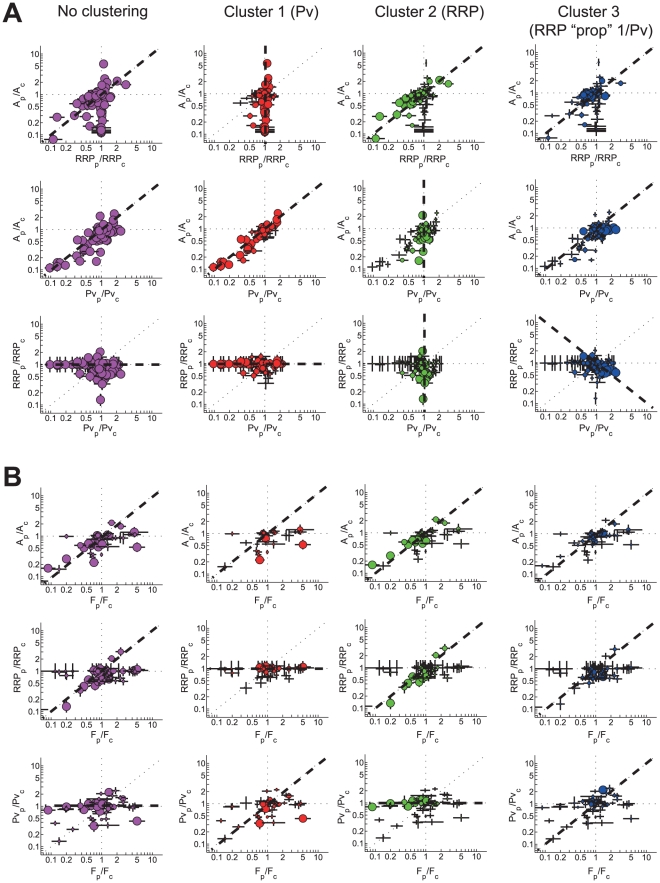
Perturbation clusters reflect distinct synaptic function. A) Functional data on synaptic vesicle release from all perturbations is plotted for the three clusters and the non-clustered case. RRP “prop” 1/P_v_ denotes the cluster with the RRP being proportionally related to the inverse of P_v_. Marker sizes indicate the cluster probability. Dotted lines indicate the best fit of the data for four possible regression curves of 0,45, 90 and 135 degrees. B) Idem as in A but now for the correlation of evoked release variables A, RRP, P_v_ with the frequency F of spontaneous release. To avoid bias towards the A–F relation of genes with many perturbations in the database, cluster probabilities are corrected for the number of perturbations per gene plotted in the graph. Final weighted probabilities are indicated by the marker sizes.

To relate each cluster of perturbations with the priming and/or fusion step in synaptic release we analyzed the orientation of the cluster unit vectors in 3D variable space (Figure S5, Suppl. Info.). This revealed that perturbations in cluster 1 were affecting P_v_, whereas perturbations in cluster 2 were involved in RRP. Furthermore, an interesting negative correlation between P_v_ and RRP was found for perturbations in cluster 3. These findings were confirmed by analysis of the orientation of the individual perturbations in 2D. In Figure 4A all individual perturbations are plotted per cluster, with marker sizes scaled with the probability to belong to that cluster. We calculated for all variable subspaces the best fits out of four possible regression curves, which gave a significant 51% reduction of the total sum of weighted orthogonal errors compared to the non-clustered case (Figure 4A, Figure S6, Table S3, M&M Suppl. Info.). Hence, perturbation data is not randomly scattered in variable space but clustered in at least three classes with specific orientations, indicative of different synaptic functions. As a validation of our method we included in our analysis specific perturbations of *Mecp2*, *Psen* and *App* that were reported to affect RRP by changing the number of synapses, whereas other perturbations in the database did not affect synapse number. Although these perturbations had either positive or negative effects on synapse number, all were assigned correctly to the RRP cluster.

### Perturbations of the same gene associate with multiple release steps

**Figure 5:**
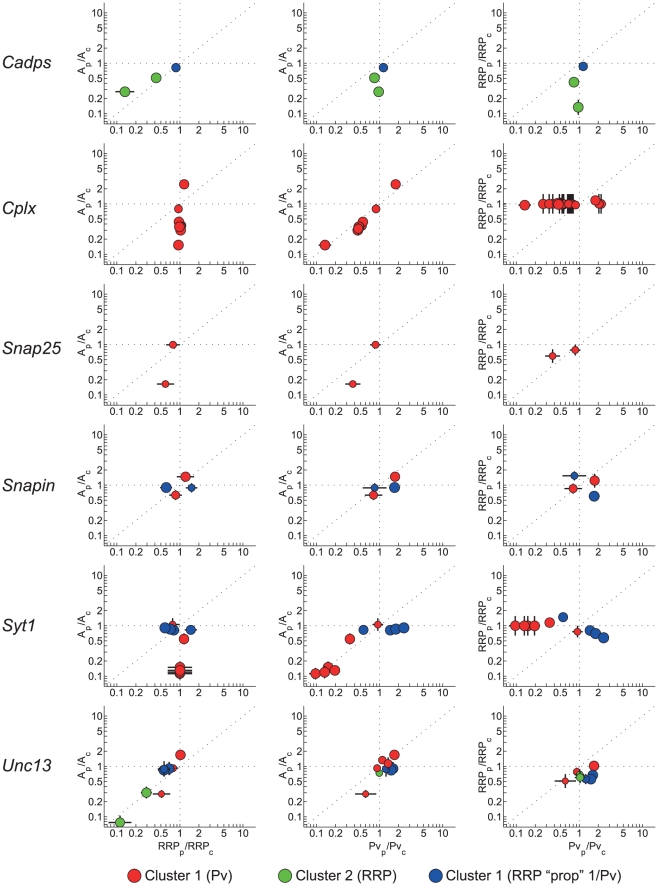
Perturbations from the same gene are assigned to different clusters. Perturbations are plotted per gene with marker color indicating to which cluster the perturbation is assigned and marker size indicating its cluster probability.

Next we addressed the question to what extent presynaptic genes are involved in multiple steps in release. We analyzed how different perturbations of the same gene were distributed over the three clusters for 9 genes that had one or more perturbations reported in the database, including those from isoforms (Figure 5, Table S4). Three genes had all their perturbations assigned to one cluster (*Cplx* and *Snap25* to the P_v_ cluster, *Mecp2* to the RRP cluster). However, the other 6 genes (*Cadps*, *Psen*, *Snapin*, *Stxbp*, *Syt* and *Unc13*) had their perturbations distributed over different clusters. This was most prominent for *Snapin*, *Syt1*, and *Unc13*, with 4 or more perturbations in the database, of which more than one third was assigned to a different cluster than their preferred cluster. Close inspection showed that most of the individual perturbations for these genes were highly significant in the original papers and tended to cluster with high specificity (P>0.8). Additional entropy analysis showed that indeed their distribution over different clusters was not due to weak classification (i.e. aspecific clustering) (Suppl. Info, Table S4). This confirms the idea that genes are involved in different steps in release, with individual perturbations having distinct functional effects. Homeostatic mechanisms, in which disturbance of synaptic transmission by genetic perturbations lead to the expression of other synaptic gene products to normalize the release process, could be an alternative explanation for these findings. However, studies in our database that measured protein levels reported these to be unchanged or mildly reduced (interpreted as decreased stability of direct binding partners) [13], [15], [16], [19], [20]. Most likely synaptic protein complexes operate at locations too remote from the cell nucleus to enable such direct feedback loops.

Studies in other model systems on individual genes seem to support this finding with reported effects of *Syt1* on RRP, P_v_, positional priming, and docking, *Snapin* on RRP and synchronous fusion, *Stxbp1* on docking, priming and PDBU dependent potentiation, and *Unc13a* on priming and PDBU dependent potentiation [21], [22], [23], [24], [25], [26]. A picture emerges where some genes have a central role in the release machinery and are involved in multiple steps, whereas others, such as *Cplx*, act as modifiers of a specific step in the release cycle. These modifying genes have possibly evolved to fine-tune the specific behavior of different types of synapses. In this context it is interesting to see that in the case of *Cplx* different isoforms across species can have an opposite effect on release [27], [28].

### Gain-of-function perturbations affect RRP inversely with P_v_

We noted that a relatively large number of the perturbations in the RRP “prop” 1/P_v_ cluster was designed to enhance neurotransmission by introducing gain-of-function (GOF) mutants (for instance enhanced calcium sensitivity mutants of *Syt1* and PDBU activation mimicking mutants of *Unc13a*, see Table S2). To test if GOF perturbations were significantly more assigned to the RRP “prop” 1/P_v_ cluster we used a weighted contingency analysis to compare how GOF perturbations and loss-of-function (LOF) perturbations were distributed over the three clusters. Indeed, GOF perturbations did significantly cluster more in the RRP “prop” 1/P_v_ cluster, with an average probability of 0.86, which was corrected for a possible bias from multiple GOF perturbations per gene (Table S5, Figure S7). In contrast, the LOF perturbations had no significant preference for any cluster. The inverse relation between the change in P_v_ and RRP for GOF mutants is in line with the idea that these proteins are involved both in priming and fusion. Mutating a protein domain to enhance one function might change the protein structure such that another function becomes impaired. Similarly, LOF mutations targeted to one function might be not specific as well. This would explain why some LOF perturbations with large effect size do not have a clear preference for one cluster, reflecting a mixed P_v_ and RRP phenotype. This suggests that the presynaptic protein network has evolved as a highly integrated molecular machine, with a group of core proteins that is involved in multiple steps of release. Indeed, biochemical evidence shows a strongly connected network with protein interactions between many presynaptic core proteins [29], [30]. Furthermore, it was shown that PKC-dependent and -independent pathways in the synapse are interdependent and need to be activated simultaneously to induce presynaptic plasticity [31].

### Same set of presynaptic genes controls spontaneous and evoked release

Finally, we investigated whether genes that control AP induced release are also involved in spontaneous release which is relevant in a long-standing debate on whether or not evoked and spontaneous release share the same vesicle pools and synaptic pathways [32], [33], [34], [35], [36], [37]. In all 3 clusters and the non-clustered situation, the 45° regression curve yields the best fit of the A–F correlation indicating that the majority of the perturbations affecting AP induced release also affect spontaneous release proportionally (Figure 4B, Table S3). This is confirmed by the fact that in the RRP cluster, F correlates well with RRP, but not with P_v_, implying that spontaneous release and evoked release are to a large extent driven by the same RRP-modifying genes, whereas for the P_v_ cluster the opposite holds. The fact that the same set of presynaptic genes controls spontaneous and evoked release favors a model where both modes of release are governed by the same molecular release machinery instead of being recruited from different vesicle pools. Until now the only exception seems to be *Doc2* that most likely competes with *Syt1* for snare binding but exclusively regulates spontaneous release in hippocampal neurons [38]. It should be noted however, that by some unknown mechanism some genetic perturbations affect spontaneous release differentially in autaptic cultures compared to other model systems, especially for *Syt1* and *Cplx*, which seem to act as a clamp on spontaneous release in these systems [16], [22], [39], [40], [41].

### Molecular machines in the synapse

Our novel approach to cluster non-standardized functional data from different perturbation studies allowed us to study the role of presynaptic genes in vesicle release at the network level, which could not be addressed in the original papers. We show that individual presynaptic proteins are not restricted to a single step in the release process but have evolved to contribute to multiple steps. Currently there is too little physical interaction data available, for instance from immunoprecipitation or yeast-two-hybrid experiments, to get a complete picture of the presynaptic protein interactome. This makes it difficult to interpret our results in terms of molecular interactions in the network, although we do know that protein complexes are formed and play an important role in the vesicle release machinery [10], [29], [30], [42]. We would like to discuss three network scenarios for the presynapse, and to what extend they can explain our findings. The first option is that the proteins in presynaptic terminal form a highly connected network, with a high number of cross-reactions, and many proteins involved in many steps of the vesicle release cycle. This seems unlikely since most protein networks have a scale-free organization, which implies that clusters of locally connected proteins are connected with each other through a few hub nodes in the network [43]. A second possibility is that there is a single macro-molecular complex, with many presynaptic proteins directly bound to each other, which is involved in the steps of docking, priming and fusion. However, there is no biochemical evidence for such a large complex in the presynapse and it would be difficult to reconcile with the observation that many genetic perturbations affect exclusively one step only. Finally, a third possibility is that the presynaptic release machinery comprises multiple smaller and dynamic protein complexes, which adapt their molecular configuration and function as the vesicles progress in the vesicle cycle. In this view, the stereotypical presynaptic protein complex does not exist. Instead, a whole range of complexes can be formed around a core set of proteins, with their specific function determined by different combinations of proteins or isoforms. For instance, different C2 domain proteins could compete for binding to the SNARE complex and make the vesicles more prone for spontaneous or action potential evoked release [44]. Likewise, a particular protein can be part of different complex configurations associated with different steps. For example, binding of Syt1 to Stx1/SNAP25 complexes is important for vesicle docking whereas Syt1 also functions as a calcium sensor for synchronous release when bound to the fully assembled SNARE complex [25], [45]. The fact that some Syt1 gain-of-function mutants both affect priming and fusion, whereas the Syt1 KO only shows a strong reduction of P_v_, could be explained by partial redundancy of the system: *i.e.* other proteins can take over the role of Syt1 in docking/priming, but not in vesicular release probability, while the Syt1 mutants have a dominant negative effect.

Our method for probabilistic clustering of genetic perturbation data can in principle be applied to any high dimensional functional dataset obtained by medium or low-throughput experiments, including imaging or behavioral data. The success of this method will be enhanced through the availability of standardized and well-annotated functional data in publicly accessible databases, which are expected to become more and more available in the coming years.

## Methods

See the supplementary material (Text S1) for a detailed description of the materials and methods.

## Supporting Information

Figure S1 Pearson pairwise correlations among the functional variables from the 35 control experiments. The panels on the diagonal show a histogram of each of the four functional variables. The off-diagonal panels show the pairwise correlations. In each panel we report the number of variables (because of missing values this number varies), the Pearson correlation coefficient and the p-value of a linear regression (from an F-test). A and P_v_ showed a correlation which was significant after Bonferroni correction for multiple comparisons (p<0.0083). Correlation for other variable combinations was not significant.
(PDF)

Figure S2 Analysis of correlations between experimental conditions (salt concentrations and days in vitro) on the functional variables from the 35 control experiments. In each panel we report the number of variables (because of missing values this number varies), the Pearson correlation coefficient and the p-value of the correlation.
(PDF)

Figure S3 Cluster assignment probabilities q_kj_ for all 121 perturbations. Red = probability of belonging to P_v_ cluster, green = probability of belonging to RRP cluster, blue = probability of belonging to RRP “prop” 1/P_v_ cluster. Perturbations are ordered with increasing cluster assignment probability for the dominant cluster. This ordering is roughly the same as the ordering of the average co-occurrence matrix. Row and column number into the co-occurrence matrix are indicated after the underscore.
(PDF)

Figure S4 Cluster assignment probability entropies E_j_ as a function of RMS perturbation distance from control condition RMS_j_. The color coding of the dots is determined by the cluster for which the perturbation has the largest assignment probability q_kj_.
(PDF)

Figure S5Cluster unit vectors 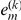 show separation of perturbation clusters A) Crosses: fitted unit vectors plotted in the 3D evoked release variable space. Solid black curve (great circle) indicates all possible functional variable combinations obeying the release model. Open circles denote the projection of each fitted unit vector onto the release model circle. Red = P_v_ cluster, green = RRP cluster, blue = RRP “prop” 1/P_v_ cluster B) Orthographic projection of the unit sphere on the RRP-P_v_ variable subspace. Solid black ellipse is the projection of the release model curve. Crosses and open circles as in panel A.(PDF)

Figure S6 Blue: average orthogonal error of the proportional linear model of 1000 random permutations of the data matrix *X*. Red line: observed average orthogonal error of the proportional linear model as plotted in Figure 4A.
(PDF)

Figure S7 Gain-of-function perturbations affect RRP inversely with P_v_. Weighted contingency analysis shows a significant higher prevalence of gain-of-function perturbations in the RRP “prop” 1/P_v_ cluster than loss-of-function perturbations. See text for explanation of the randomization test based on the Pearson Chi square statistic.
(PDF)

Table S1 Excel file with presynaptic gene database: Column description: (A) name of first author on publication, (B) Pubmed ID, (C) abbreviated NCBI name of the perturbed gene or applied compound, (D) alias used in the publication if different from the NCBI gene name, (E) full NCBI gene name, (F) name of the gene family to which the perturbed gene (isoform) belongs, (G) description of the perturbation or control group, (H) number of rows above the current row to which perturbation data should be normalized, (I) Indicate WT data (1 data set per publication) for the analysis of experimental variability between studies (1 if only A is reported, 2 if both A and RRP are reported), (J-M) salt concentrations in mM used in extracellular medium, (N) ratio of extracellular calcium and magnesium, (O) additional compounds in extracellular medium, (P) age of measured cells expressed in number of days in vitro (DIV), (Q) average DIV, (R) range of DIV, (S) temperature at which the experiments were performed, (T) description of the experimental groups with reference to the data source in the publication, (U) mean amplitude expressed in nA unless otherwise indicated in the previous column, (V) SEM, (W) number of measurements, (X-AA) idem for RRP (in pC unless otherwise indicated) as in column T-W, (AB-AE) idem for P_v_ (%), (AF-AI) idem for F (in Hz unless otherwise indicated).
(XLS)

Table S2 Excel file with clustering results for 121 genetic perturbations: file contains sheets sorted by gene name, cluster, and author. Column description: (A) name of first author on publication, (B) Pubmed ID, (C) abbreviated NCBI name of the perturbed gene or applied compound, (D) alias used in the publication if different from the NCBI gene name, (E) full NCBI gene name, (F) name of the gene family to which the perturbed gene (isoform) belongs, (G) description of the perturbation, (H) description of the control group to which the perturbation is normalized, (I) row index in the presynaptic gene database (Table S1) of the perturbation, (J) row index in the presynaptic gene database of the control group, (K) index of perturbation in the co-occurrence matrix (Figure 3), (L-O) synaptic variables normalized to control, (P-W) log2 transformed normalized synaptic variables with log2 transformed SEM of the normalized variables, (X-Z) probabilities for clusters 1–3, (AB) entropy measure for cluster specificity calculated from the cluster probabilities using eq. 13, (AD) RMS calculated from the log2 transformed normalized data using eq. 12.
(XLS)

Table S3 Excel file with correlation model fits: (A) Weighted orthogonal errors (not corrected and corrected for the average probability) for fits to the different correlation models (0, 45, 90, 135 degrees) in the 2D projections of the evoked release variable space plotted in Figure 4A. The average probability of all data points shown in a particular subspace (this may vary between subspaces because not all variables are reported for all subspaces) are given for each subspace and cluster. (B) Idem for the correlation between the spontaneous release variable F and the evoked release variables as plotted in Figure 4B.
(XLS)

Table S4 Excel file with the average cluster probabilities per gene calculated from the probabilities of the individual perturbations.
(XLS)

Table S5 Selection of Gain-of-Function mutants (GOF) and Loss-of-Function mutants (LOF) for contingency analysis in Figure S7.
(XLS)

Text S1 Supplementary info with extended Materials and Methods.
(PDF)

## References

[1] Abbott LF, Regehr WG (2004). Synaptic computation.. Nature.

[2] Chua JJ, Kindler S, Boyken J, Jahn R (2010). The architecture of an excitatory synapse.. J Cell Sci.

[3] Emes RD, Pocklington AJ, Anderson CN, Bayes A, Collins MO (2008). Evolutionary expansion and anatomical specialization of synapse proteome complexity.. Nat Neurosci.

[4] Sudhof TC (2004). The synaptic vesicle cycle.. Annu Rev Neurosci.

[5] Giaever G, Chu AM, Ni L, Connelly C, Riles L (2002). Functional profiling of the Saccharomyces cerevisiae genome.. Nature.

[6] Ohya Y, Sese J, Yukawa M, Sano F, Nakatani Y (2005). High-dimensional and large-scale phenotyping of yeast mutants.. Proc Natl Acad Sci U S A.

[7] Fraser AG, Kamath RS, Zipperlen P, Martinez-Campos M, Sohrmann M (2000). Functional genomic analysis of C. elegans chromosome I by systematic RNA interference.. Nature.

[8] Lee SS, Lee RY, Fraser AG, Kamath RS, Ahringer J (2003). A systematic RNAi screen identifies a critical role for mitochondria in C. elegans longevity.. Nat Genet.

[9] Boutros M, Kiger AA, Armknecht S, Kerr K, Hild M (2004). Genome-wide RNAi analysis of growth and viability in Drosophila cells.. Science.

[10] Rizo J, Rosenmund C (2008). Synaptic vesicle fusion.. Nat Struct Mol Biol.

[11] Takamori S, Holt M, Stenius K, Lemke EA, Gronborg M (2006). Molecular anatomy of a trafficking organelle.. Cell.

[12] Schneggenburger R, Sakaba T, Neher E (2002). Vesicle pools and short-term synaptic depression: lessons from a large synapse.. Trends Neurosci.

[13] Augustin I, Rosenmund C, Sudhof TC, Brose N (1999). Munc13-1 is essential for fusion competence of glutamatergic synaptic vesicles.. Nature.

[14] Fernandez-Chacon R, Konigstorfer A, Gerber SH, Garcia J, Matos MF (2001). Synaptotagmin I functions as a calcium regulator of release probability.. Nature.

[15] Jockusch WJ, Speidel D, Sigler A, Sorensen JB, Varoqueaux F (2007). CAPS-1 and CAPS-2 are essential synaptic vesicle priming proteins.. Cell.

[16] Reim K, Mansour M, Varoqueaux F, McMahon HT, Sudhof TC (2001). Complexins regulate a late step in Ca2+-dependent neurotransmitter release.. Cell.

[17] Bekkers JM, Stevens CF (1991). Excitatory and inhibitory autaptic currents in isolated hippocampal neurons maintained in cell culture.. Proc Natl Acad Sci U S A.

[18] Ikeda K, Bekkers JM (2006). Autapses.. Curr Biol.

[19] Toonen RF, Wierda K, Sons MS, de Wit H, Cornelisse LN (2006). Munc18-1 expression levels control synapse recovery by regulating readily releasable pool size.. Proc Natl Acad Sci U S A.

[20] Geppert M, Goda Y, Hammer RE, Li C, Rosahl TW (1994). Synaptotagmin I: a major Ca2+ sensor for transmitter release at a central synapse.. Cell.

[21] Dulubova I, Lou X, Lu J, Huryeva I, Alam A (2005). A Munc13/RIM/Rab3 tripartite complex: from priming to plasticity?. EMBO J.

[22] Liu H, Dean C, Arthur CP, Dong M, Chapman ER (2009). Autapses and networks of hippocampal neurons exhibit distinct synaptic transmission phenotypes in the absence of synaptotagmin I.. J Neurosci.

[23] Lou X, Korogod N, Brose N, Schneggenburger R (2008). Phorbol esters modulate spontaneous and Ca2+-evoked transmitter release via acting on both Munc13 and protein kinase C.. J Neurosci.

[24] Young SM, Neher E (2009). Synaptotagmin has an essential function in synaptic vesicle positioning for synchronous release in addition to its role as a calcium sensor.. Neuron.

[25] de Wit H, Walter AM, Milosevic I, Gulyas-Kovacs A, Riedel D (2009). Synaptotagmin-1 docks secretory vesicles to syntaxin-1/SNAP-25 acceptor complexes.. Cell.

[26] Gulyas-Kovacs A, de Wit H, Milosevic I, Kochubey O, Toonen R (2007). Munc18-1: sequential interactions with the fusion machinery stimulate vesicle docking and priming.. J Neurosci.

[27] Cho RW, Song Y, Littleton JT (2010). Comparative analysis of Drosophila and mammalian complexins as fusion clamps and facilitators of neurotransmitter release.. Mol Cell Neurosci.

[28] Xue M, Reim K, Chen X, Chao HT, Deng H (2007). Distinct domains of complexin I differentially regulate neurotransmitter release.. Nat Struct Mol Biol.

[29] Schoch S, Gundelfinger ED (2006). Molecular organization of the presynaptic active zone.. Cell Tissue Res.

[30] Wang X, Hu B, Zieba A, Neumann NG, Kasper-Sonnenberg M (2009). A protein interaction node at the neurotransmitter release site: domains of Aczonin/Piccolo, Bassoon, CAST, and rim converge on the N-terminal domain of Munc13-1.. J Neurosci.

[31] Wierda KD, Toonen RF, de Wit H, Brussaard AB, Verhage M (2007). Interdependence of PKC-dependent and PKC-independent pathways for presynaptic plasticity.. Neuron.

[32] Fredj NB, Burrone J (2009). A resting pool of vesicles is responsible for spontaneous vesicle fusion at the synapse.. Nat Neurosci.

[33] Groemer TW, Klingauf J (2007). Synaptic vesicles recycling spontaneously and during activity belong to the same vesicle pool.. Nat Neurosci.

[34] Ikeda K, Bekkers JM (2009). Counting the number of releasable synaptic vesicles in a presynaptic terminal.. Proc Natl Acad Sci U S A.

[35] Mathew SS, Pozzo-Miller L, Hablitz JJ (2008). Kainate modulates presynaptic GABA release from two vesicle pools.. J Neurosci.

[36] Sara Y, Virmani T, Deak F, Liu X, Kavalali ET (2005). An isolated pool of vesicles recycles at rest and drives spontaneous neurotransmission.. Neuron.

[37] Hua Z, Leal-Ortiz S, Foss SM, Waites CL, Garner CC (2011). v-SNARE composition distinguishes synaptic vesicle pools.. Neuron.

[38] Groffen AJ, Martens S, Diez Arazola R, Cornelisse LN, Lozovaya N (2010). Doc2b is a high-affinity Ca2+ sensor for spontaneous neurotransmitter release.. Science.

[39] Xue M, Lin YQ, Pan H, Reim K, Deng H (2009). Tilting the balance between facilitatory and inhibitory functions of mammalian and Drosophila Complexins orchestrates synaptic vesicle exocytosis.. Neuron.

[40] Maximov A, Tang J, Yang X, Pang ZP, Sudhof TC (2009). Complexin controls the force transfer from SNARE complexes to membranes in fusion.. Science.

[41] Huntwork S, Littleton JT (2007). A complexin fusion clamp regulates spontaneous neurotransmitter release and synaptic growth.. Nat Neurosci.

[42] Sorensen JB (2009). Conflicting views on the membrane fusion machinery and the fusion pore.. Annu Rev Cell Dev Biol.

[43] Vazquez A, Alzate O (2010). Protein Interaction Networks.. Neuroproteomics.

[44] Walter AM, Groffen AJ, Sorensen JB, Verhage M (2011). Multiple Ca2+ sensors in secretion: teammates, competitors or autocrats?. Trends Neurosci.

[45] Kochubey O, Schneggenburger R (2011). Synaptotagmin increases the dynamic range of synapses by driving Ca(2)+-evoked release and by clamping a near-linear remaining Ca(2)+ sensor.. Neuron.

