# Supplementary figures and images for: Molecular Machines in the Synapse: Overlapping Protein Sets Control Distinct Steps in Neurosecretion
Source: PLoS Comput Biol. 2012 Apr 5;8(4):e1002450. doi: 10.1371/journal.pcbi.1002450 (PMC3320570; doi:10.1371/journal.pcbi.1002450)

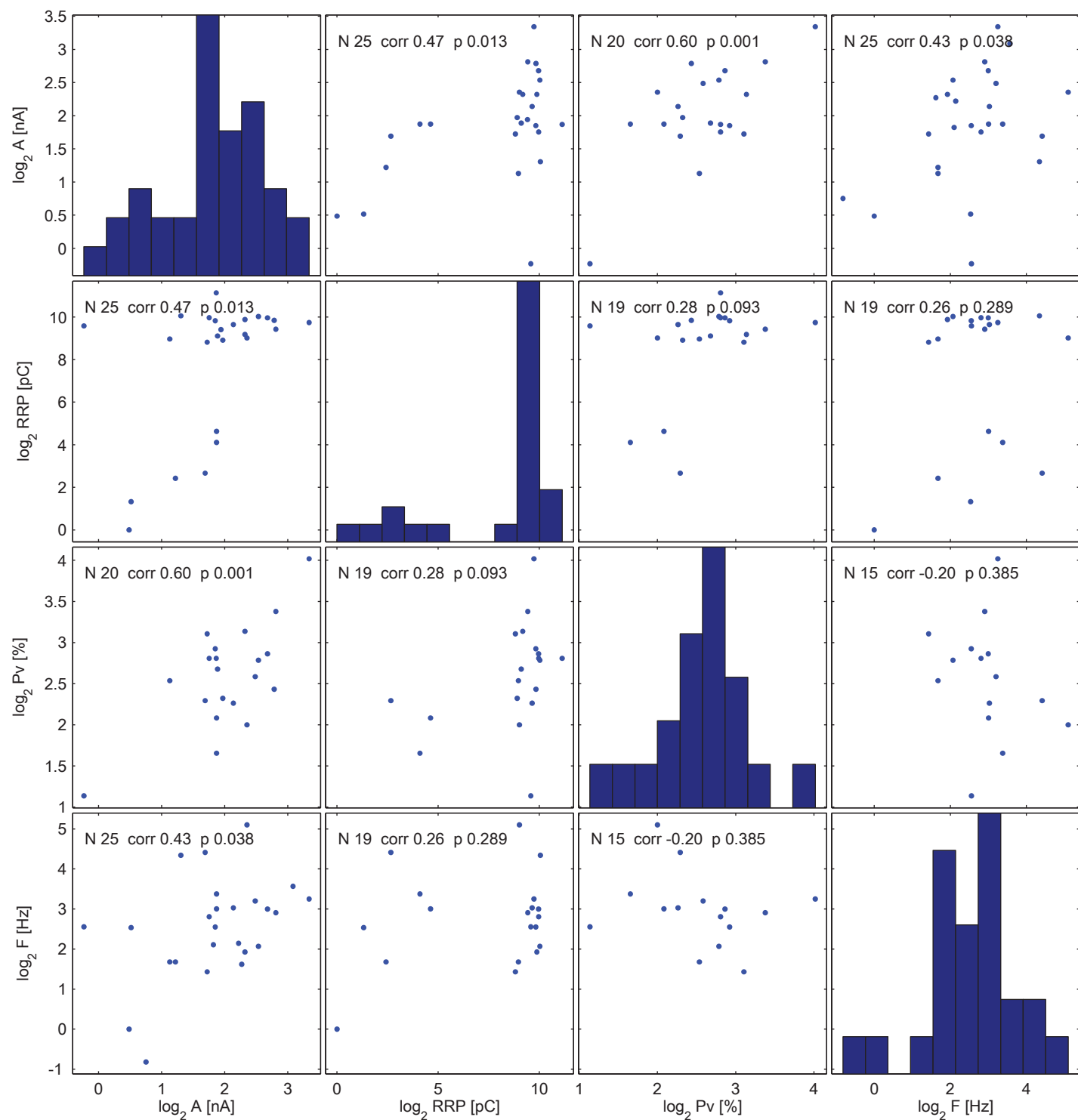

Figure S1 Cornelisse et al.

Supplement: Figure S1 — Pearson pairwise correlations among the functional variables from the 35 control experiments. The panels on the diagonal show a histogram of each of the four functional variables. The off-diagonal panels show the pairwise correlations. In each panel we report the number of variables (because of missing values this number varies), the Pearson correlation coefficient and the p-value of a linear regression (from an F-test). A and Pv showed a correlation which was significant after Bonferroni correction for multiple comparisons (p<0.0083). Correlation for other variable combinations was not significant. (PDF) [file pcbi.1002450.s001.pdf]

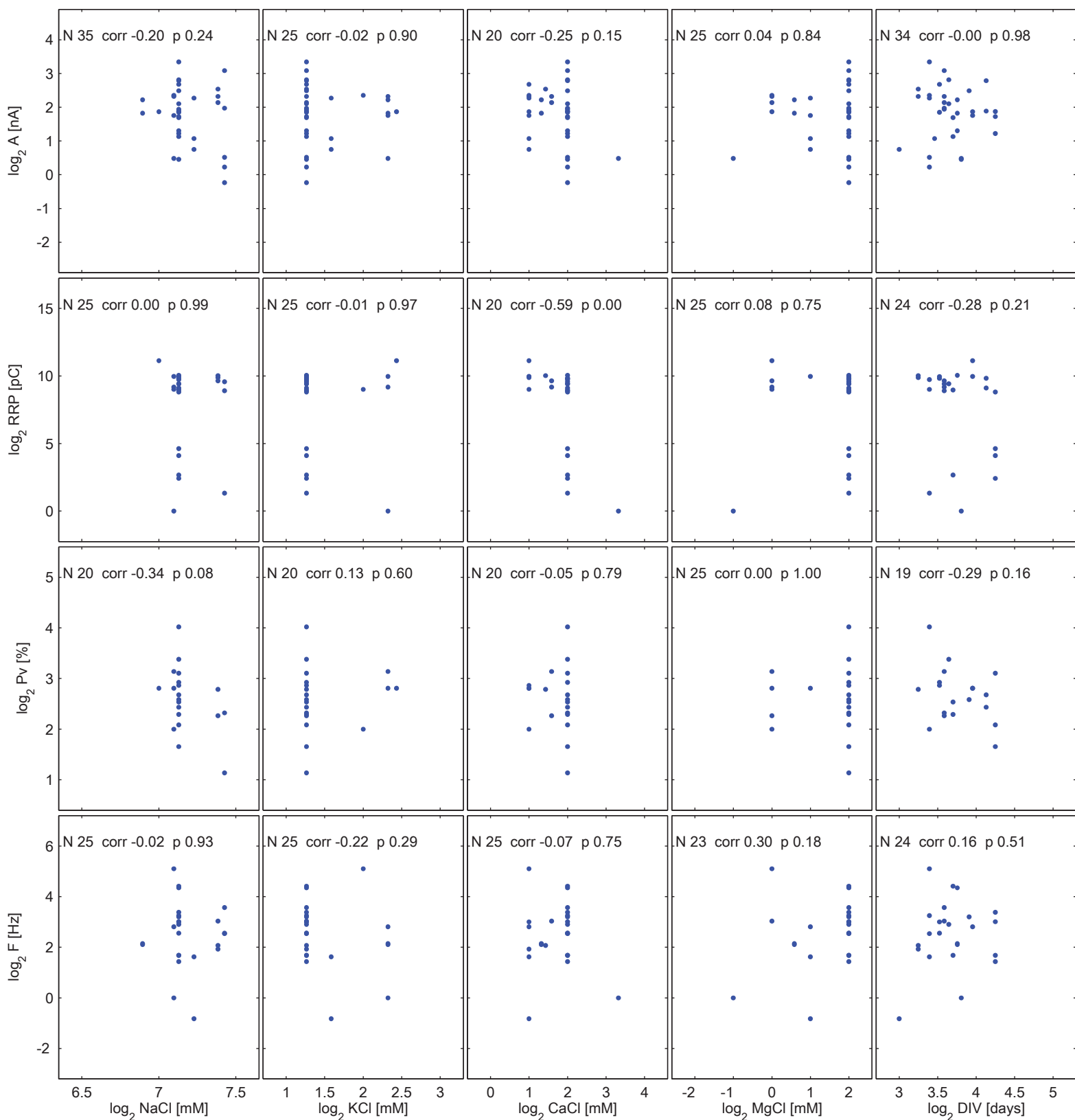

Figure S2 Cornelisse et al.

Supplement: Figure S2 — Analysis of correlations between experimental conditions (salt concentrations and days in vitro) on the functional variables from the 35 control experiments. In each panel we report the number of variables (because of missing values this number varies), the Pearson correlation coefficient and the p-value of the correlation. (PDF) [file pcbi.1002450.s002.pdf]

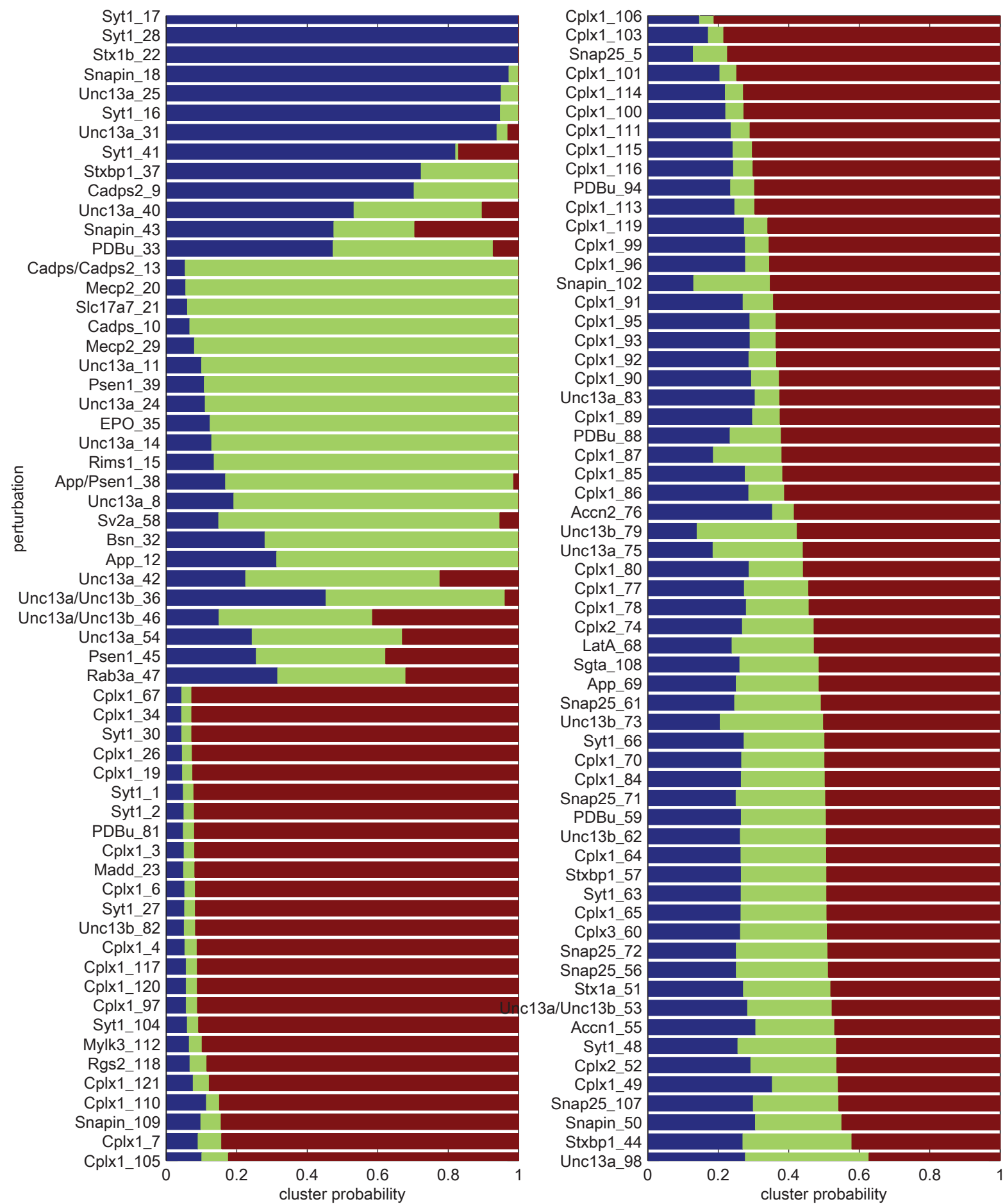

Figure S3 Cornelisse et al.

Supplement: Figure S3 — Cluster assignment probabilities qkj for all 121 perturbations. Red = probability of belonging to Pv cluster, green = probability of belonging to RRP cluster, blue = probability of belonging to RRP “prop” 1/Pv cluster. Perturbations are ordered with increasing cluster assignment probability for the dominant cluster. This ordering is roughly the same as the ordering of the average co-occurrence matrix. Row and column number into the co-occurrence matrix are indicated after the underscore. (PDF) [file pcbi.1002450.s003.pdf]

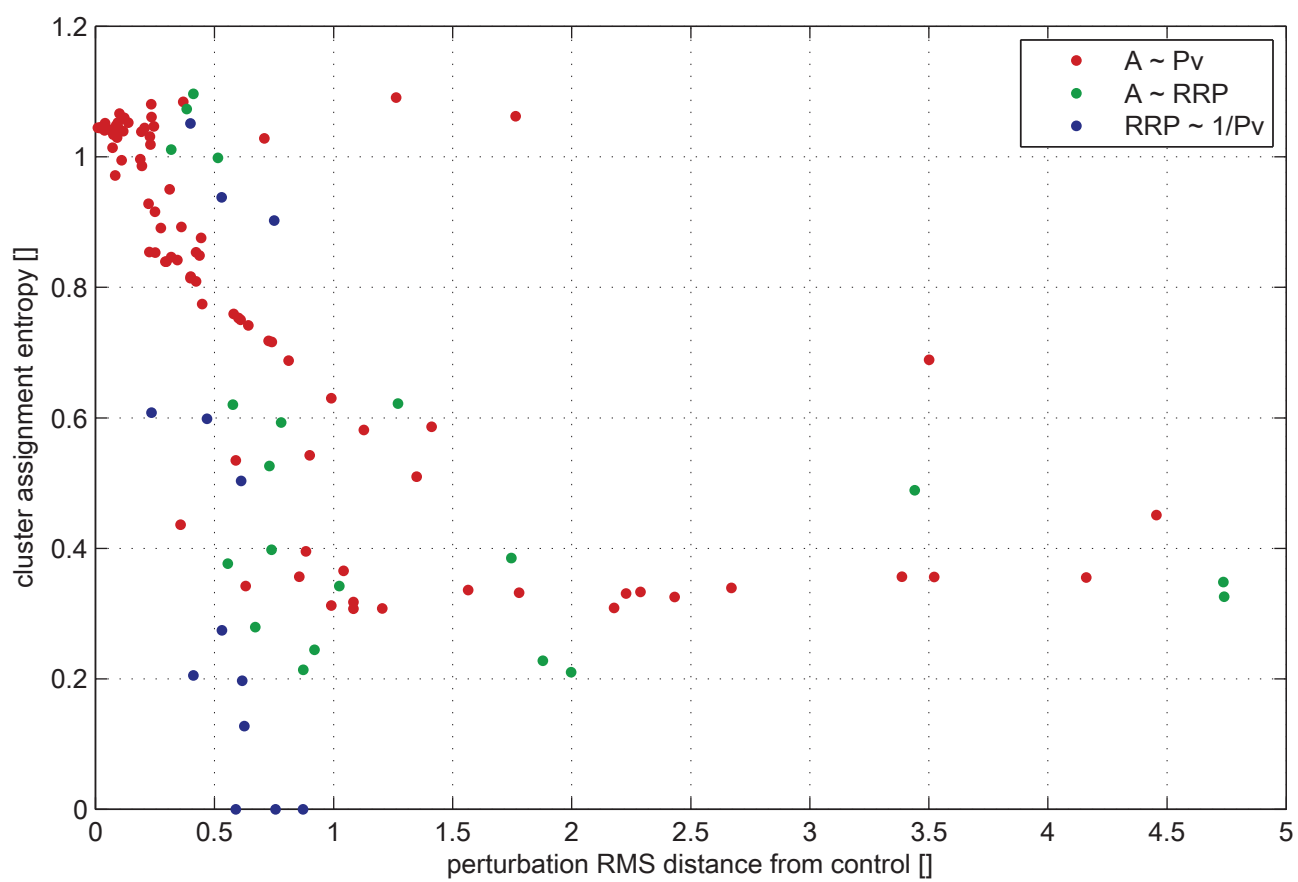

Figure S4 Cornelisse et al.

Supplement: Figure S4 — Cluster assignment probability entropies Ej as a function of RMS perturbation distance from control condition RMSj. The color coding of the dots is determined by the cluster for which the perturbation has the largest assignment probability qkj. (PDF) [file pcbi.1002450.s004.pdf]

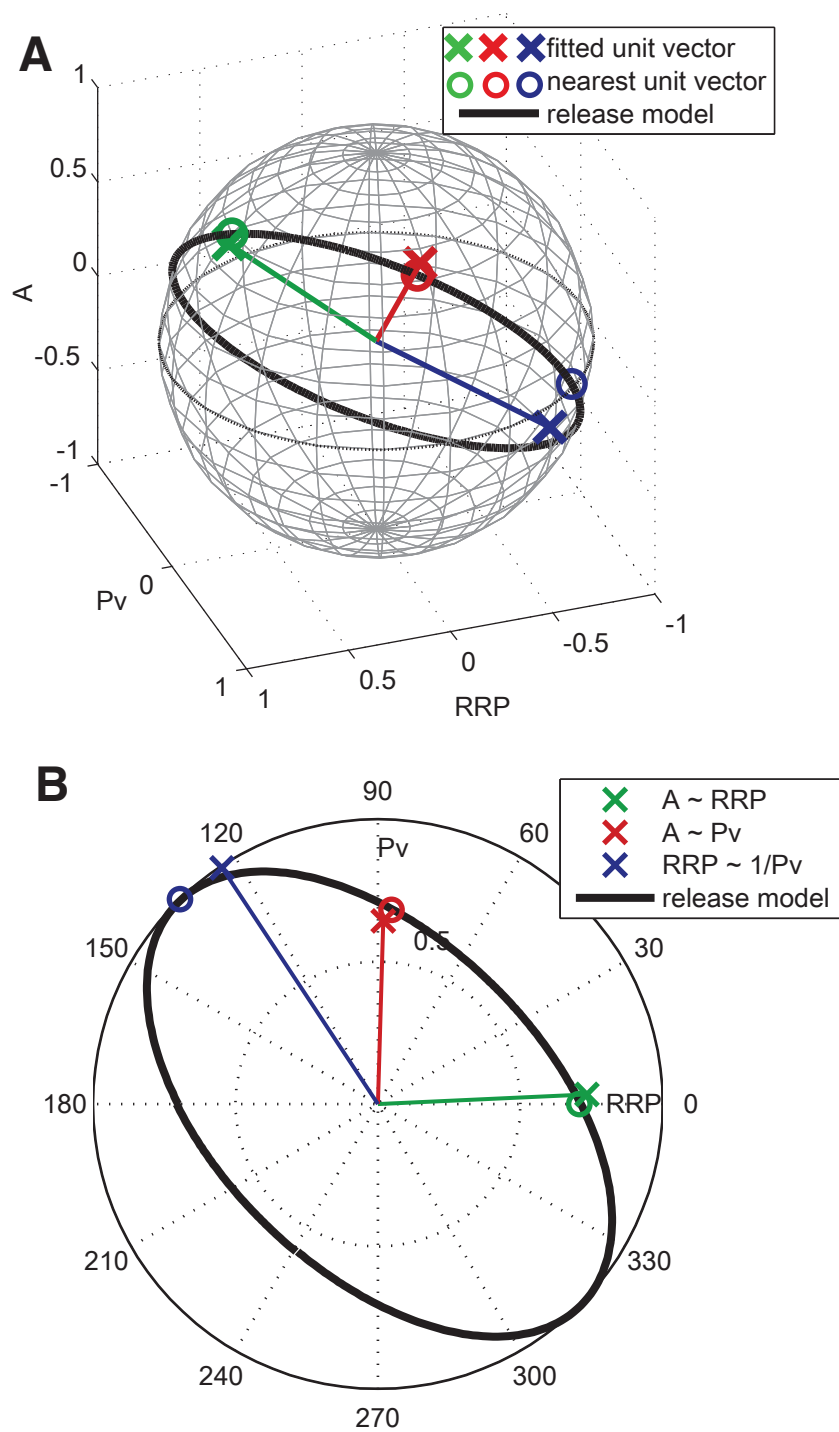

Figure S5 Cornelisse et al.

Supplement: Figure S5 — Cluster unit vectors show separation of perturbation clusters A) Crosses: fitted unit vectors plotted in the 3D evoked release variable space. Solid black curve (great circle) indicates all possible functional variable combinations obeying the release model. Open circles denote the projection of each fitted unit vector onto the release model circle. Red = Pv cluster, green = RRP cluster, blue = RRP “prop” 1/Pv cluster B) Orthographic projection of the unit sphere on the RRP-Pv variable subspace. Solid black ellipse is the projection of the release model curve. Crosses and open circles as in panel A. (PDF) [file pcbi.1002450.s005.pdf]

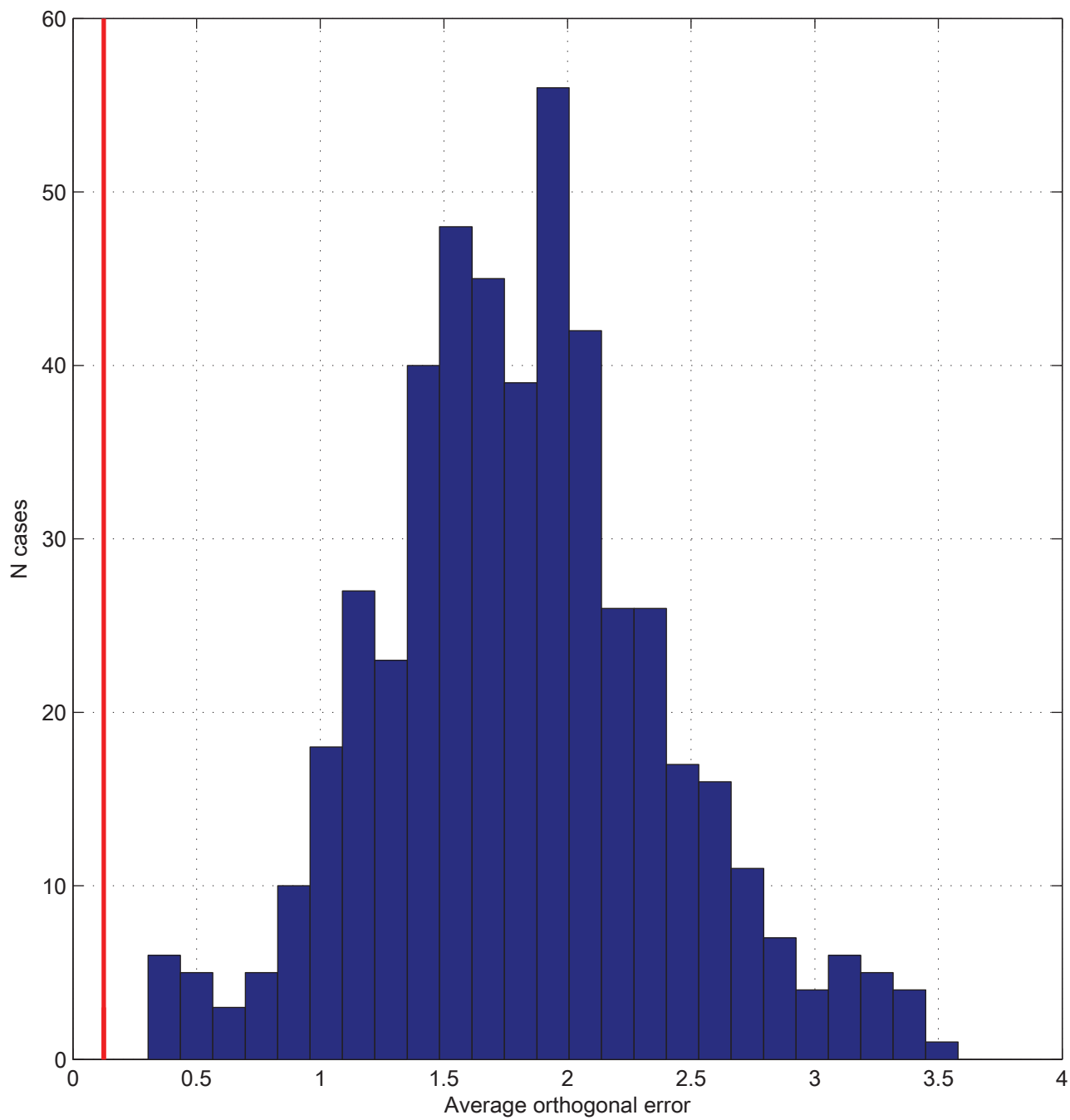

Figure S6 Cornelisse et al.

Supplement: Figure S6 — Blue: average orthogonal error of the proportional linear model of 1000 random permutations of the data matrix X. Red line: observed average orthogonal error of the proportional linear model as plotted in Figure 4A. (PDF) [file pcbi.1002450.s006.pdf]

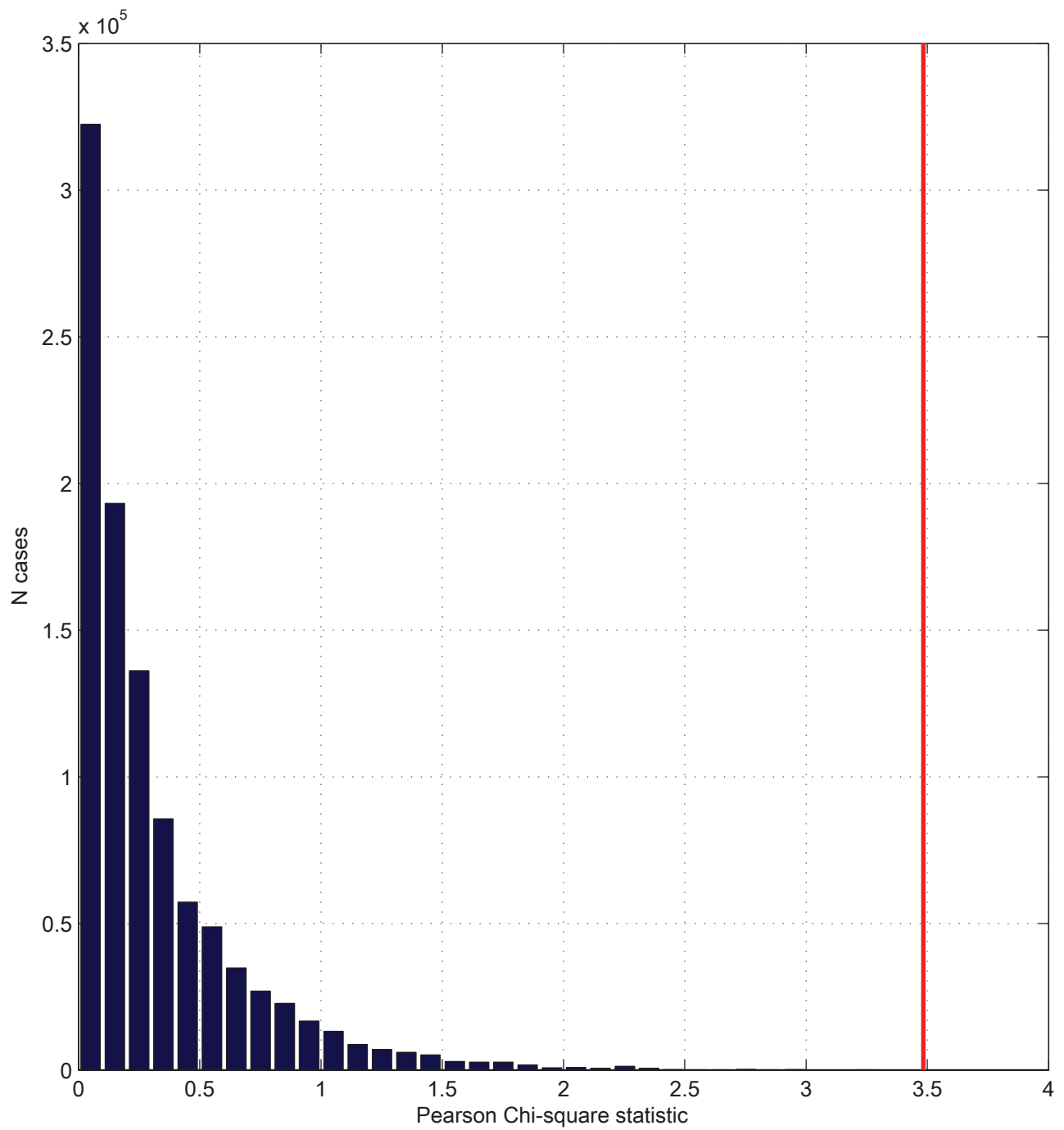

Figure S7 Cornelisse et al.

Supplement: Figure S7 — Gain-of-function perturbations affect RRP inversely with Pv. Weighted contingency analysis shows a significant higher prevalence of gain-of-function perturbations in the RRP “prop” 1/Pv cluster than loss-of-function perturbations. See text for explanation of the randomization test based on the Pearson Chi square statistic. (PDF) [file pcbi.1002450.s007.pdf]
