# Supplementary material for: Molecular Machines in the Synapse: Overlapping Protein Sets Control Distinct Steps in Neurosecretion
Source: PLoS Comput Biol. 2012 Apr 5;8(4):e1002450. doi: 10.1371/journal.pcbi.1002450 (PMC3320570; doi:10.1371/journal.pcbi.1002450)
Supplement: Text S1 — Supplementary info with extended Materials and Methods. (PDF) [file pcbi.1002450.s013.pdf]

# Supplementary Information for "Molecular machines in the synapse: overlapping protein sets control distinct steps in neurosecretion"

L. Niels Cornelisse[1], Evgeni Tsivtsivadze[2], Marieke Meijer[1],
Tjeerd M.H. Dijkstra[2,3], Tom Heskes[2], Matthijs Verhage[1]

[1] Functional Genomics Center for Neurogenomics and Cognitive Research (CNCR), FALW-VUA,
Dept. of Clinical Genetics, VUmc, Neuroscience Campus Amsterdam (NCA),
VU University (VUA) and VU University medical center (VUmc), The Netherlands
[2] Machine Learning Group, Inst. for Computing and Information Sciences,
Radboud University Nijmegen, The Netherlands
[3] Signal Processing Systems, Dept of Electrical Engineering,
Technical University Eindhoven, The Netherlands

10 February 2012

## Contents

**1 Materials and methods** . . . . . **1**
- 1.1 Data collection . . . . . 1
- 1.2 Clustering . . . . . 2
  - 1.2.1 Model constraints . . . . . 2
  - 1.2.2 Analysis of toy data to gain intuition in model . . . . . 3
  - 1.2.3 Preprocessing . . . . . 3
  - 1.2.4 From release model to principal component analysis . . . . . 4
  - 1.2.5 Probabilistic principal component analysis . . . . . 5
  - 1.2.6 A mixture of probabilistic PCA for clustering perturbations . . . . . 6
  - 1.2.7 Consensus clustering for the assignment probabilities . . . . . 7
- 1.3 Entropy and perturbation size . . . . . 8
  - 1.3.1 Analysis of perturbation size and entropy of cluster assignment probability . . . . . 8
  - 1.3.2 Entropy analysis to quantify cluster specificity . . . . . 8
- 1.4 Analysis of unit vectors . . . . . 9
- 1.5 Analysis of pairwise proportionality with orthogonal least squares . . . . . 9
- 1.6 Contingency analysis of gain-of-function vs loss-of-function mutants . . . . . 10

## 1 Materials and methods

### 1.1 Data collection

We retrieved functional data on synaptic transmission from cultured autaptic neurons. This preparation allows versatile assessment of synaptic variables from a large, well-defined population of synapses and is also an ideal and widely applied preparation to analyze genetic perturbations using neurons from mutant mice or by applying

viral constructs. A perturbation was defined as a pharmacological or genetic manipulation, and included gene knock-out (KO), knock-in (KI), gene knock down (KD), and overexpression of a wild-type (WT) or mutant variant on a WT or KO background (OE).

In this study the following variables were included:

A Amplitude of the evoked excitatory post synaptic current (EPSC), as a measure of evoked synaptic release (Figure $1C_1$)

RRP Readily releasable pool defined by the integral of the postsynaptic current response to a 500 mOsmol hypertonic sucrose application, reporting vesicle priming at steady state (Figure $1C_2$)

$P_v$ Vesicular release probability, reflecting the effectiveness of vesicle fusing during action potential stimulation. $P_v$ is a composite variable, obtained from the linear relation in the release model (eq. 1) between release (R) and the product of RRP and $P_v$ by dividing the total EPSC charge, obtained by integration of the EPSC, by the total charge of the RRP.

F Frequency of spontaneous release events (miniature EPSC's or mEPSC's) (Figure $1C_3$).

All variables are expressed as mean and standard error of the mean (SEM) and stored in an Excel database (Microsoft, Redmond, USA) after being text mined from literature or in some cases reconstructed from graphs using in-house written software in Matlab (The Mathworks, Natick, USA). The data sources are indicated in column 1 of table S1. In a few cases, when SEMs were not reported, the SEM for perturbation $j$, functional variable $m$ was conservatively estimated as:

$$s_{jm} = 2x_{jm} \frac{\sum_{j' \neq j} s_{j'm}}{\sum_{j' \neq j} x_{j'm}}$$

Thus, missing SEMs are imputed from twice the average weighted SEM of the other perturbations. Some variables were reported as normalized to the control group. Since our normalization algorithm in the preprocessing step (see below) normalizes by dividing through the variable value in the control group we imputed value 1 for the corresponding variables in the control group.

## 1.2 Clustering

### 1.2.1 Model constraints

We designed the data analysis model with the following aims: (1) as the amplitude of the genetic perturbation is difficult to control, the results should not depend on the size of the perturbation (2) the model should take into account the variability of the data (standard errors as reported in the studies where the data were taken from) and (3) the model should be able to deal with missing data (as most studies only report a subset of observables). A mixture of probabilistic principal component analyzers (MPPCA) fulfills these constraints as detailed below. Effectively, the algorithm clusters the orientations of the functional data, weighted by their standard errors and with missing data receiving a weight of zero.

Note that we do not exclude perturbations from the clustering algorithm based on statistical significance. Thus, some of the perturbations in the database are not significantly different from the control condition (see Table S4 for an analysis of significance of selected perturbations based on the t-test). These individually non-significant perturbations will still contribute to the overall clustering. This is a general property of

statistical models in that they integrate weak data to support an overall stronger conclusion. Also note that the majority of small perturbations are assigned with roughly equal probability to each of the clusters, see Figure S4.

Our MPPCA differs from an earlier approach by Tipping and Bishop [6], as they do not deal with input with specified standard errors nor with missing data. Also, Tipping and Bishop estimate multiple principal components whereas we only employ the first one. As we only use the first principal component our algorithm could also be termed a "mixture of weighted orthogonal least squares regressors" but we prefer the MPPCA moniker.

### 1.2.2 Analysis of toy data to gain intuition in model

To obtain intuition about the model we analyze a toy data set by hand (in section 1.2.6 we give the mathematical details of the full model). For simplicity, we take the toy data (1) to obey the release model perfectly, (2) to come from two clusters, one where amplitude A is proportional to RRP and one where A is proportional to $P_v$ and (3) one positive and one negative perturbation relative to control for a total of four perturbations. Writing the four perturbations as row vectors with the elements in the row vector in the order A-RRP-$P_v$, we have as toy data: $\mathbf{x}_1 = (2, 2, 0)$, $\mathbf{x}_2 = (2, 0, 2)$, $\mathbf{x}_3 = (-2, -2, 0)$ and $\mathbf{x}_4 = (-2, 0, -2)$. By design, perturbations 1 and 3 belong to cluster 2 (see Figure 4 for definition of clusters) and perturbations 2 and 4 to cluster 1. From the MPPCA model we get the following output: first the cluster assignment probabilities $q$ are given by $q_1 = q_3 = (0, 1)$ and $q_2 = q_4 = (1, 0)$ with the first element of each $q_j$ denoting the probability of belonging to cluster 1 and the second element the probability of belonging to cluster 2. As the toy data was designed to fit perfectly in a cluster, the probabilities are 1 (for the right cluster) or 0 (for the wrong cluster). From the cluster assignment probabilities we get the co-occurence matrix as (see eq. 11):

$$C = \begin{pmatrix} 1 & 0 & 1 & 0 \\ 0 & 1 & 0 & 1 \\ 1 & 0 & 1 & 0 \\ 0 & 1 & 0 & 1 \end{pmatrix}.$$

The co-occurence matrix $C$ shows that perturbations 1-3 and 2-4 always cooccur hence are in the same cluster. By reordering the rows and columns as in Figure 2 we get:

$$C_{\text{reordered}} = \begin{pmatrix} 1 & 1 & 0 & 0 \\ 1 & 1 & 0 & 0 \\ 0 & 0 & 1 & 1 \\ 0 & 0 & 1 & 1 \end{pmatrix}$$

Second, the unit vectors are given by $\mathbf{e}^{(1)} = (1/\sqrt{2}, 0, 1/\sqrt{2})$ and $\mathbf{e}^{(2)} = (1/\sqrt{2}, 1/\sqrt{2}, 0)$. Third, the model perturbation amplitudes are given by $\mathbf{a}_1 = (0, 2\sqrt{2})$, $\mathbf{a}_2 = (2\sqrt{2}, 0)$, $\mathbf{a}_3 = (0, -2\sqrt{2})$ and $\mathbf{a}_4 = (-2\sqrt{2}, 0)$ with the first element of each vector the model perturbation amplitude in cluster 1 and the second element in cluster 2. The model perturbation amplitudes are a nuisance parameters as it is not yet possible to control the phenotypic effect of a genetic perturbation and hence we do not report them in this study.

### 1.2.3 Preprocessing

In a minority of the studies all four variables A, RRP, $P_v$ and F were reported, but in most cases one or more variables were missing. Since A is related to RRP times $P_v$,

a minimum of two out of these three variables is sufficient to infer the effect of a perturbation on vesicle priming and fusion. All perturbations that met this criterion were included in the database, resulting in 121 perturbations from genetic manipulations of 29 single genes and 4 gene pairs, and pharmacological manipulations with 2 biochemical agents. For most genes only one or two perturbations were reported in the literature except for *Cplx1* (43), *Unc13a* (12), *Snap25* (6), *Snapin* (5), and *Syt1* (12).

Before the clustering algorithm we normalized and log-transformed the raw perturbation values, both means and SEMs. In detail we took each perturbation with non-zero value for column "normalized to n rows above" from table S1 and normalized that value to the relevant control condition as indicated by this column. Denoting with $z_{jm}$ the $j$-th perturbation of the $m$-th functional variable (with $m \in (1,2,3,4)$) and with $z_{j0}$ the relevant control condition, each perturbation mean $x_{jm}$ was calculated as:

$$x_{jm} = \log_2 z_{jm} - \log_2 z_{j0}$$

We calculated the normalized and log transformed perturbation variances $v_{jm}$ from the raw perturbation standard errors of the mean $s_{jm}$ as listed in table S1 by propagation of errors as follows:

$$v_{jm} = \left( \left( \frac{s_{jm}}{z_{jm}} \right)^2 + \left( \frac{s_{j0}}{z_{j0}} \right)^2 \right) / (\log 2)^2$$

#### 1.2.4 From release model to principal component analysis

The release model of presynaptic vesicle release relates the amplitude of evoked release ($A$) to the size of the readily releasable pool ($RRP$) and the probability of evoked release ($P_v$):

$$A = \alpha \, RRP \, P_v \tag{1}$$

with $\alpha$ a constant of proportionality. Extending the notation to include multiple perturbations, indexed with subscript $_j$, we rewrite this model as:

$$A_j = \alpha \, R_j \, p_j$$

with index $j \in (0,\ldots,J)$ with $J$ the number of perturbations and $j = 0$ the control condition (wild type). We changed the symbols for the readily releasable pool and the release probability such that each variable is represented by a single symbol (we need to add subscripts later on). In the next step, we normalize all variables relative to wild type and take logarithms, leading to:

$$A_j = R_j + p_j \tag{2}$$

where – with slight abuse of notation – each variable $Z$ is calculated from $Z_j = \log_2 Z_j - \log_2 Z_0$ with $j \in (1,\ldots,J)$. In order to get a homogeneous notation for the variables and to be able to extend the set of variables in the future, we relabel the set of variables $(A_j, R_j, p_j)$ as $x_{jm}$ with $m$ indexing the variable, $m \in (1,2,3)$. With this change, we now have a single data matrix $X$ of dimensions $J$ by 3. The release model can be rewritten in terms of the $x_{jm}$ as:

$$x_{j1} = x_{j2} + x_{j3} \tag{3}$$

and can be viewed as a linear dependency among the columns of data matrix $X$. Put another way, a singular value decomposition (SVD) of $X$ has one singular value equal to zero. However, the case where one of the singular values is equal to zero presents

a minimum as a perturbation might not affect all of $x_{j1}$, $x_{j2}$ and $x_{j3}$ but might affect only a subset of these three. For example if the probability of evoked release is not affected by perturbation j we also have the constraint $x_{j3} = 0$. Hence we can have a "mix and match" of constraints depending on the effect of each perturbation on the observed variables. Since we want to capture all these constraints in a simple model, we propose a principal component analysis (PCA) model as follows:

$$x_{jm} = a_j \, e_m \, , \tag{4}$$

with $a_j$ the perturbation specific part and $e_m$ the variable specific part. Since multiplying each $a_j$ with a factor $c$ and dividing $e_m$ with the same factor $c$ leads to the same $x_{jm}$, we normalize **e** to unit length to resolve the non-identifyability of the model. Substitution of eq. 4 in eq. 3 shows that the release model leads to a linear dependence among the $e_m$:

$$e_1 = e_2 + e_3$$

However, as stated above, there could be more constraints depending on the effect of the perturbation. Thus, the PCA model of eq. 4 can be viewed as an encompassing model assumption that incorporates all possible linear constraints among the components of vector **e**. Perturbations obeying different constraints should be classified in different clusters. In order to fit **e** from the data we first design a probabilistic version of PCA and subsequently introduce a mixture of these PPCA's to cluster similar **e**'s.

### 1.2.5 Probabilistic principal component analysis

The functional data have two characteristics that necessitate a generalization of model assumption eq. 4: variables are reported with variability estimates (calculated from repeated measurements over cells) and there are missing data (as authors did not report all variables). Let us consider all perturbation experiments and corresponding observations $x_{jm}$ and variance estimates $v_{jm}$. Our modeling assumption is

$$x_{jm} = a_j e_m + \epsilon \, , \tag{5}$$

where $\epsilon$ is normally distributed noise with precision $w_{jm}$, i.e., standard deviation $w_{jm}^{-1/2}$ obtained from the reported error estimates. When parameter $m$ is missing in perturbation experiment $j$, we simply set $w_{jm} = 0$ (and can set $x_{jm}$ to any value we like: it will not affect any of the equations). We assume that the noises for different perturbations are independent which seems reasonable as these are measured in different experiments with different samples.

Thus, we assume that the change induced by a particular perturbation factorizes into a perturbation specific part $a_j$ (e.g., over-expression, knock-out) and a variable specific part $e_m$. This is very similar to a (weighted) singular value decomposition, in which we are only interested in the largest singular value. Standard singular value decomposition, however, does not apply when we have different precisions $w_{jm}$. The model (5) is sometimes also referred to as a separable model. We can fit the parameters **a** and **e** of this model by maximizing the log likelihood of the data $X$ given these model parameters:

$$L(X, W|\mathbf{e}, \mathbf{a}) = -\frac{1}{2} \sum_{j,m} \left( w_{jm}(x_{jm} - a_j e_m)^2 - \log\left[\frac{w_{jm}}{2\pi}\right] \right) \, , \tag{6}$$

where $X$ and $W$ refer to the mean and variance data respectively. Setting the derivative w.r.t. **a** to zero and solving for **a** gives

$$a_j = \frac{\sum_m w_{jm} x_{jm} e_m}{\sum_m w_{jm} e_m^2} \, , \tag{7}$$

and doing the same for $\mathbf{e}$ gives

$$e_m = \frac{\sum_j a_j w_{jm} x_{jm}}{\sum_j a_j^2 w_{jm}} . \tag{8}$$

Iterating these two equations corresponds to coordinate-wise ascent: with each step the log likelihood increases until a (local) maximum has been found. The model is invariant under multiplication of $\mathbf{a}$ by $c$ and division of $\mathbf{e}$ by $c$. For easier interpretation, we normalize $\mathbf{e}$ to a unit vector after each update. When all precisions are equal, the log likelihood has a single global maximum (ignoring invariances as the ones just explained) which corresponds to the truncated singular value decomposition of the data matrix $X$ and thus relates to the principal component of its covariance matrix.

### 1.2.6 A mixture of probabilistic PCA for clustering perturbations

We expand the above by introducing clusters of perturbations. We assume that perturbations belonging to the same cluster share the parameter-dependent part $\mathbf{e}$ in model (5). We write $\mathbf{e}^{(k)}$ for the unit vector corresponding to cluster $k$, with $k = 1 \ldots K$, $p_k$ for the prior probability that any perturbation belongs to cluster $k$, and $\mathbf{a}^{(k)}$ for the perturbation-dependent term in model (5) when corresponding to cluster $k$. Our goal will be to fit these unit vectors, perturbation-dependent terms, and prior probabilities to the data. The log likelihood of the data given all model parameters reads:

$$\mathcal{L}(X, W|\mathbf{e}, \mathbf{a}, \mathbf{p}) = \log \sum_k p_k \exp\left[L(X, W|\mathbf{e}^{(k)}, \mathbf{a}^{(k)})\right]$$

with $L(X, W|\mathbf{e}^{(k)}, \mathbf{a}^{(k)})$ from (6).

It is relatively straightforward to derive an expectation-maximization algorithm for the maximization of this log likelihood. We introduce an auxiliary matrix $Q$ with *cluster assignment probabilities* $q_{kj}$ and write

$$\mathcal{L}(X, W|\mathbf{e}, \mathbf{a}, \mathbf{p}) = \max_Q \mathcal{F}(\mathbf{e}, \mathbf{a}, \mathbf{p}, Q|X, W) ,$$

with

$$\mathcal{F}(\mathbf{e}, \mathbf{a}, \mathbf{p}, Q|X, W) \equiv \sum_{k,j} q_{kj} L(X_{j.}, W_{j.}|\mathbf{e}^{(k)}, a_j^{(k)}) - \sum_{k,j} q_{kj} \log\left[\frac{q_{kj}}{p_k}\right] ,$$

and where the maximum is taken under the constraint $\sum_k q_{kj} = 1 \ \forall_j$. The EM-algorithm performs coordinate-wise ascent on the function $\mathcal{F}$. Hence the *mixture of probabilistic principal component analyzers* (MPPCA) algorithm consists of the following iterations:

**E-step:** Fix $\mathbf{e}$ and $\mathbf{p}$ and maximize w.r.t. $\mathbf{a}$ and then $Q$, yielding the updates:

$$\begin{aligned} a_j^{(k)} &= \frac{\sum_m w_{jm} x_{jm} e_m^{(k)}}{\sum_m w_{jm} (e_m^{(k)})^2} \\ q_{kj} &\propto p_k \exp\left[-\frac{1}{2}\sum_m w_{jm}\left(x_{jm} - a_j^{(k)} e_m^{(k)}\right)^2\right] , \end{aligned} \tag{9}$$

where the $q$'s have to be normalized such that $\sum_k q_{kj} = 1 \ \forall_j$.

**M-step:** Fix $\mathbf{a}$ and $Q$ and maximize w.r.t. $\mathbf{e}$ and $\mathbf{p}$, yielding the updates

$$\begin{aligned} e_m^{(k)} &= \frac{\sum_j q_{kj} a_j^{(k)} w_{jm} x_{jm}}{\sum_j q_{kj} (a_j^{(k)})^2 w_{jm}} \\ p_k &\propto \sum_j q_{kj} , \end{aligned} \tag{10}$$

where $\mathbf{p}$ has to be normalized such that $\sum_k p_k = 1$ and each $\mathbf{e}^{(k)}$ is normalized to $|\mathbf{e}^{(k)}| = 1$.

To summarize the algorithm, inputs are the $J$ by $M$ dimensional data matrices $X$ and $W$ and the number of clusters $K$. Output consists of (1) a $K$ by $J$ dimensional cluster assignment matrix $Q$, (2) $K$ by $M$ dimensional unit vectors $\mathbf{e}^{(k)}$ and $K$ $J$ dimensional perturbation vectors $\mathbf{a}^{(k)}$.

To complete the discussion of the algorithm, we discuss the starting and ending conditions. To start the iterations, we initialize $p_k = 1/K$ and $\mathbf{e}^{(k)}$ to a random sample from a normal distribution with zero mean and unit variance. Each $\mathbf{e}^{(k)}$ is normalized to unit length. As the model log likelihood $\mathcal{L}$ depends on the starting condition, we typically run the algorithm multiple times and keep results from the run with the highest model log likelihood.

As stopping criterion we use a combination of the change in unit vector and assignment matrix. As results hardly depend on the details of the stopping criterion we do not detail this any further.

### 1.2.7 Consensus clustering for the assignment probabilities

The assignment probabilities computed by our algorithm depend on the starting condition and the number of clusters. To make our approach robust we run the basic MPPCA algorithm in two nested loops and average the assignment probabilities. The inner loop runs for 100 times and is designed to exclude local maxima. Only the ten runs with the highest model log likelihood $\mathcal{L}$ are kept. The outer loop is designed to vary the cluster number $K$. We varied $K$ between 3 and 12 (Figure 3). After running the algorithm with $R = 100$ different restarts and number of clusters we obtain soft assignment matrices $Q_1, \ldots, Q_R$. We average the assignment probabilities in a co-occurrence matrix $C$ [4]. Each entry $C_{jl}$ indicates the probability that over $R$ runs two perturbations $j$ and $l$ occur in the same cluster. We define it as follows:

$$C_{jl} = \frac{1}{R} \sum_{k=1}^{K} \sum_{r=1}^{R} q_{kj}^{(r)} q_{kl}^{(r)} . \tag{11}$$

or better visualization we order all co-occurrence matrices using the algorithm described in [2]. To obtain robust cluster probabilities, we decomposed the consensus co-occurrence matrix $C$ by fuzzy additive clustering into $J$ consensus assignment probabilities $\mathbf{r}_j$ ([3]). Each of the $J$ $\mathbf{r}_j$ is a 3-dimensional vector giving the assignment probability to belong to each of the three clusters. The consensus assignment probabilities are computed by minimizing the following loss function:

$$f(\mathbf{R}) = \sum_{j=1}^{J} \sum_{l=j+1}^{J} (C_{jl} - \mathbf{r}_j^T \mathbf{r}_l)^2,$$

with $\mathbf{R}$ denoting the $J$ by $K$ matrix with columns $\mathbf{r}_j$, see Figure S3. We solved the optimization problem by iterative row-wise quadratic programming as suggested in

[3]. As both the co-occurrence matrices for each cluster number as the consensus one showed three noticeable clusters (Figure 3), we used $J = 3$ clusters for this final step of our procedure.

## 1.3 Entropy and perturbation size

### 1.3.1 Analysis of perturbation size and entropy of cluster assignment probability

We hypothesize that larger perturbations (further away from the control condition) are more likely to fall in a single cluster. To quantify the "size" of a perturbation we used the root-mean-square (RMS) as this measure is insensitive to missing data. We calculated the RMS for perturbation $j$ from the data matrix $X$ of dimension $J = 121$ by 3 with:

$$RMS_j = \sqrt{\frac{1}{M_j^*} \sum_{m=1}^{M_j^*} x_{jm}^2}, \tag{12}$$

with index $m$ running over the non-missing data and $M_j^*$ denoting the number of non-missing variables. To quantify how strongly a perturbation was clustered into one of the three clusters we used the entropy defined as:

$$E_j = \sum_{k=1}^{3} -q_{kj} \log q_{kj}, \tag{13}$$

with log denoting the natural logarithm. In Figure S4 we plot $E_j$ as a function of $\text{RMS}_j$ confirming our hypothesis that larger perturbations are more likely to be assigned to a single cluster. For the analysis whether perturbations of a gene were assigned to different clusters we excluded perturbations with small perturbation size (RMS $< 0.2$) since these tended to distribute evenly over the clusters due to their large variance.

### 1.3.2 Entropy analysis to quantify cluster specificity

Since we used a "soft" probabilistic approach the distributed clustering of perturbations of the same gene could be due to either non-specific clustering with more or less evenly distributed cluster probabilities, or specific clustering with assignment of perturbations to distinct clusters with high probability. The first scenario suggests a gene with a unique function that clusters poorly with other genes. The second scenario reflects a gene involved in different steps in release with individual perturbations affecting a single step only.

In order to quantify the cluster specificity of multiple perturbations $j$ from gene $i$ we compared the mean entropy (ME), defined as the average of the entropies $E_j$ of the individual perturbations, with the entropy of the mean (EOM), calculated using eq. 13, with $q_{kj} = \text{mean}(q_{kj}^{(i)})$, the average cluster probabilities for the perturbations of gene $i$. By construction, EOM $\geq$ ME but if EOM $\sim$ ME the individual perturbations are clustered similarly. However, if EOM $\gg$ ME individual perturbations are assigned to different clusters with high probability.

Whereas genes with all perturbations in one cluster showed a small increase of the entropy of the mean (EOM) compared to the mean entropy (ME) (Cplx 8%, Mecp2 0.5%, Snap25 5%), this increase was much larger for genes with perturbations assigned to multiple clusters (Cadps 66%, Psen 28%, Snapin 53%, Stxbp1 19%, Syt 147%, Unc13 55%), indicating that for these genes cluster specificity was high.

## 1.4 Analysis of unit vectors

We observed that the unit vectors vary little between restarts with the same cluster number $K$. Hence, the unit vectors reported in Figure S5 are from the run of the basic MPPCA algorithm with $K = 3$ with the highest log likelihood.

The release model can be viewed as a constraint on the possible unit vectors, that lie on the unit sphere spanned by the functional variables A, RRP and $P_v$. Geometrically, the locus of unit vectors consistent with the release model consists of the intersection of the plane defined by eq. 2 with the unit sphere. Introducing longitude $0 \leq \phi < 2\pi$ and co-latitude $-\pi/2 \leq \theta \leq \pi/2$ we define:

$$\begin{aligned} A &= \sin\theta \\ R &= \cos\theta\cos\phi \\ p &= \cos\theta\sin\phi \end{aligned}$$

Combining this with eq. 2 we get for the locus of points on the unit vector sphere consistent with the release model:

$$\tan\theta = \cos\phi + \sin\phi,$$

which describes a great circle on the unit sphere. In orthographic projection (viewing the unit sphere down the A axis), this great circle becomes an ellipse with a long axis at orientation 135 degrees relative to the RRP-axis and an aspect ratio of $1/\sqrt{3}$ (Figure S5).

## 1.5 Analysis of pairwise proportionality with orthogonal least squares

In order to interpret the clusters using the assignment probabilities we fitted four proportional linear models (Figure 4). Denoting the functional variable plotted on the horizontal axis with $x$ and the one plotted on the vertical axis with $y$, the four models are $y = -x$, $y = 0$, $y = x$ and $x = 0$. Given a set of data points $(x_j, y_j)$ we pick the model with the smallest orthogonal distance. We discuss this first for the "no clustering" case, left-hand column of subplots in Figure 4A. The orthogonal distance of each data point relative to each of the four models is given by:

$$\begin{aligned} D_{j,y=-x} &= |x_j + y_j|/\sqrt{2} \\ D_{j,y=0} &= |y_j| \\ D_{j,y=x} &= |x_j - y_j|/\sqrt{2} \\ D_{j,x=0} &= |x_j|. \end{aligned}$$

Using $m \in (1, \ldots, 4)$ to index the models, the best model minimizing the sum of distances from each data point, is given by:

$$m^* = \underset{m}{\operatorname{argmin}} \sum_{j=1}^{J} D_j^{(m)}. \tag{14}$$

In the other three columns of Figure 4A, we weigh each orthogonal distance with cluster assignment probabilities $r_{kj}$ normalized for the average cluster assignment probability. In detail:

$$\begin{aligned} r_{kj} &= \frac{q_{kj}}{\sum_{j=1}^{J} q_{kj}} \\ m_A^*(k) &= \underset{m}{\operatorname{argmin}} \sum_{j=1}^{J} r_{kj}\, D_j^{(m)}, \end{aligned}$$

for each of the three clusters $k \in (1, \ldots, 3)$.

In Figure 4B, we include the frequency of spontaneous release $F$ in the analysis. Just as in the left-hand panels of Figure 4A, in the left-hand panels of Figure 4B, we use the unweighted distance of eq. 14. In the other three columns of Figure 4B, we weight each orthogonal distance with cluster assignment probabilities $t_{kj}$ normalized for the number of perturbations per gene, denoted by $N_j^{(i)}$. In detail:

$$
\begin{aligned}
t_{kj} &= \frac{q_{kj}}{N_j^{(i)}} \\
m_B^*(k) &= \operatorname*{argmin}_m \sum_{j=1}^{J} t_{kj}\, D_j^{(m)},
\end{aligned}
$$

for each of the three clusters $k \in (1, \ldots, 3)$.

To validate whether the proportional linear models capture the information contained in the dataset we conduct the following analysis. Given the input data matrix $x_{jm}$ we randomly permute its columns and rows. This corresponds to the situation when a normalized perturbation can have, for example, in place of functional variable A a value that corresponds to the functional variable $P_v$ of some other perturbation. Once the dataset is permuted, we perform the consensus clustering analysis and fit the four proportional linear models as described above. We repeat the randomization 1000 times and record the orthogonal error of the four models averaged over the clusters for every repetition. From this we construct the histogram depicted in Figure S6 where the vertical line corresponds to the average orthogonal error obtained from the original (not permuted) dataset. We note that the difference between the permuted and original data is statistically significant ($p < 0.001$) as none of the 1000 random permutations had a lower average orthogonal error than the original data set.

### 1.6 Contingency analysis of gain-of-function vs loss-of-function mutants

To support the idea that gain-of-function mutants are significantly more likely than loss-of-function mutants to be assigned to the RRP prop $1/P_v$ cluster, we performed a contingency analysis. Conventionally, one uses the Pearson $\chi^2$-test to investigate whether cluster assignment probabilities are independent of mutant class (gain-of-function or loss-of-function) [1]. However, the Pearson $\chi^2$-statistic is only $\chi^2$ distributed for a large number of cases, whereas we have only 12 genes. Secondly, the cluster assignment probabilities are graded not binary as in conventional contingency analysis. Thus, exact tests, like Fisher's exact test are impossible. Hence, we opted for a random permutation test as follows.

First, we averaged the assignment probabilities for those genes with more than one perturbation (see Table S5). Second, we calculated the Pearson $\chi^2$-statistic for our sample of 12 genes and found its value to be 3.48, indicated with a vertical red line in Figure S7. Third, we randomly permuted the mutant class of the genes $10^6$ times and calculated a histogram of Pearson $\chi^2$-statistic values, indicated in blue in Figure S7. Lastly, we counted the number randomly permuted Pearson $\chi^2$-statistic values larger than the observed one of 4.14 (6 values in our case). The p-value from the random permutation test is then $6\ 10^{-6}$.

## References


[1] Agresti, Categorical Data Analysis, Wiley, 2002.

[2] Bar-Joseph, Gifford and Jaakkola, Fast optimal leaf ordering for hierarchical clustering, *Bioinformatics*, 17:S22–S29, 2001.

[3] ter Braak, Kourmpetis, Kiers and Bink, Approximating a similarity matrix by a latent class model: a reappraisal of additive fuzzy clustering, *Computational Statistics and Data Analysis*, 53:3183-3193, 2009.

[4] Grotkjaer, Winther, Regenberg, Nielsen and Hansen, Robust multi-scale clustering of large DNA microarray datasets with the consensus algorithm, *Bioinformatics*, 22:58–67, 2006.

[5] Hahsler, Hornik and Buchta, Getting things in order: an introduction to the R package seriation, *Journal of Statistical Software*, 25, 2008.

[6] Tipping and Bishop, Mixtures of probabilistic principal component analyzers, *Neural Computation*, 11, 1999.
